# Experimental assessment of capacities for cumulative culture: Review and evaluation of methods

**DOI:** 10.1002/wcs.1516

**Published:** 2019-08-23

**Authors:** Christine A. Caldwell, Mark Atkinson, Kirsten H. Blakey, Juliet Dunstone, Donna Kean, Gemma Mackintosh, Elizabeth Renner, Charlotte E. H. Wilks

**Affiliations:** ^1^ Division of Psychology University of Stirling Stirling Scotland

**Keywords:** comparative psychology, cultural evolution, cumulative culture, developmental psychology

## Abstract

In the current literature, there are few experimental tests of capacities for cumulative cultural evolution in nonhuman species. There are even fewer examples of such tests in young children. This limited evidence is noteworthy given widespread interest in the apparent distinctiveness of human cumulative culture, and the potentially significant theoretical implications of identifying related capacities in nonhumans or very young children. We evaluate experimental methods upon which claims of capacities for cumulative culture, or lack thereof, have been based. Although some of the established methods (those simulating generational succession) have the potential to identify positive evidence that fulfills widely accepted definitions of cumulative culture, the implementation of these methods entails significant logistical challenges. This is particularly true for testing populations that are difficult to access in large numbers, or those not amenable to experimental control. This presents problems for generating evidence that would be sufficient to support claims of capacities for cumulative culture, and these problems are magnified for establishing convincing negative evidence. We discuss alternative approaches to assessing capacities for cumulative culture, which circumvent logistical problems associated with experimental designs involving chains of learners. By inferring the outcome of repeated transmission from the input–output response patterns of individual subjects, sample size requirements can be massively reduced. Such methods could facilitate comparisons between populations, for example, different species, or children of a range of ages. We also detail limitations and challenges of this alternative approach, and discuss potential avenues for future research.

This article is categorized under:

Cognitive Biology > Evolutionary Roots of Cognition
Cognitive Biology > Cognitive Development
Psychology > Comparative Psychology

## Graphical abstract

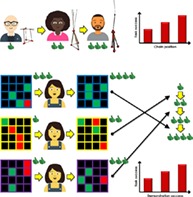

## 1 INTRODUCTION

In humans, cultural transmission can result in the accumulation of knowledge and skills over generations of transmission, such that learners in later generations are benefitted, relative to their predecessors, by virtue of their exposure to this social information. This phenomenon, usually described as cumulative culture, or cumulative cultural evolution, appears to be widespread in human populations. However, there is much debate over the extent to which similar phenomena are apparent in nonhuman species. There is also increasing interest in the question of when human children develop capacities needed to support cumulative cultural evolution.

In spite of this interest, it has proven difficult to design empirical tests which adequately assess capacities for cumulative culture. This makes it very difficult to address questions about the existence or otherwise of capacities for cumulative culture in particular populations. It is harder still to answer questions about any constraints limiting the expression of these capacities, which might explain why we see limited evidence of cumulative culture in the natural behavior of certain populations. These difficulties arise primarily because cumulative cultural evolution is inherently a group‐level process, which describes patterns of change to cultural traits which occur as a consequence of repeated transmission. Although definitions vary, cumulative culture is usually identified as a special case of cultural evolution, characterized by a directional pattern of change typically resulting in “improvements” (Tennie, Call, & Tomasello, 2009) or increasingly “preferred” traits (Caldwell, 2018). Mesoudi and Thornton (2018) recently defined four core criteria for cumulative culture, these being: (a) a change to a behavior or cultural product, (b) social transmission of the modified trait, (c) improvement in performance as a consequence of the modification, and (d) iteration of these steps resulting in ongoing improvement over time. The centrality of consistent directional change as a defining feature of cumulative culture was also captured by Tomasello's (1990) “ratchet” analogy, and the resulting description of cumulative culture as a “ratchet effect” (p. 305). As such, experiments aiming to test for the presence or otherwise of cumulative culture generally involve groups of participants, arranged into overlapping learner generations who can each learn from their immediate predecessors, with each individual attempting the same experimentally controlled task (e.g., Caldwell & Millen, 2008; Reindl & Tennie, 2018; Sasaki & Biro, 2017). The presence or otherwise of cumulative culture is then determined by the resulting trends in any changes in task success over generations. This approach has been successfully applied in studies involving adult humans (e.g., Caldwell & Millen, 2008; Osiurak et al., 2016; Zwirner & Thornton, 2015), confirming the potential for identifying cumulative culture. However, these multigenerational experimental designs are challenging to implement, and studies involving populations other than adult humans are therefore extremely scarce.

In this article we review the current state of the art in experimental research testing capacities for cumulative culture in nonhuman animals and human children. We identify challenges associated with the dominant methodological approaches, which limit these methods' potential to address key questions. We also propose a solution to these problems, describing a novel methodological approach which we believe offers great potential for tackling these same critical issues.

## 2 MOTIVATIONS FOR EXPERIMENTAL ASSESSMENT OF CAPACITIES FOR CUMULATIVE CULTURE

There are a number of reasons why researchers have been interested in the question of whether particular populations (in addition to adult humans) exhibit capacities potentially supporting cumulative culture. For example, such evidence might shed light on questions surrounding the apparent distinctiveness of human cultural traditions, compared with the socially transmitted behaviors of other species. This question has been the subject of much debate, with some theorists asserting that cumulative culture is, or is expected to be, restricted to humans alone (e.g., Tennie et al., 2009; Tomasello, Kruger, & Ratner, 1993), while others have argued for continuity, leading to claims of cumulative culture, or close approximations thereof, in a range of nonhuman species (e.g., New Caledonian crows (Hunt & Gray, 2003); baboons (Claidière, Smith, Kirby, & Fagot, 2014); pigeons (Sasaki & Biro, 2017); chimpanzees, (Vale, Davis, Lambeth, Schapiro, & Whiten, 2017); Japanese macaques, (Schofield, McGrew, Takahashi, & Hirata, 2017); bighorn sheep and moose (Jesmer et al., 2018)). Used as a diagnostic tool, adequate experimental evidence could in theory reveal the phylogenetic distribution of capacities supporting cumulative culture (as full potential will not necessarily be apparent from natural behavior, and since even the most compelling observational evidence is generally open to interpretation). Furthermore, experimentally controlled comparisons could even shed light on questions about factors constraining cumulative culture in nonhuman species.

In relation to children's capacities for cumulative culture, the issues at stake are rather different. If operating on the assumption that human adults are capable of cumulative culture, but that ratchet‐like cultural transmission is probably precluded in very young children whose social learning skills are only just developing, it therefore follows that the relevant capacities must emerge ontogenetically, during childhood. Studying children's capacities for cumulative culture thus potentially provides insights into key cognitive developments that support this process, and could provide insights into the extent to which human propensities for cumulative culture are experience‐dependent (e.g., Heyes, 2018).

## 3 ASSESSING CAPACITIES FOR CUMULATIVE CULTURE: CURRENT STATE OF THE ART

In this section we consider the evidence upon which claims of cumulative culture have been based, in nonhumans and children. We also include studies explicitly reporting absence of evidence in investigations of cumulative culture in these groups.

### 3.1 Claims based on spontaneous behavior of natural populations

It is worth noting at the outset that cultural evolution is well documented in the vocal dialects of both birds and cetaceans (e.g., Lynch & Baker, 1993; Noad, Cato, Bryden, Jenner, & Jenner, 2000). However, these vocal cultures are rarely argued to be cases of *cumulative* cultural evolution as it is unclear (as with many examples from human culture) whether the mutations represent objective “improvements” relative to the traits they replaced. There have, however, been a variety of claims of cumulative cultural evolution based on observations of wild nonhuman populations, in other behavioral domains. A number of such putative cases were reviewed by Dean, Vale, Laland, Flynn, and Kendal (2014), including examples from chimpanzees, capuchin monkeys, macaques, and crows. As noted by Dean et al. (2014), however, these claims were largely based on circumstantial evidence, with processes of gradual modification being inferred from apparently complex final forms, and/or transmission via social learning inferred from within‐group behavioral similarities. However, since then, some noteworthy claims have been made, based on substantial historical datasets.

Schofield et al. (2017) reviewed over 60 years of data from field observations of provisioned, free‐ranging Japanese macaques on the island of Koshima (e.g., Kawai, 1965). Schofield et al. (2017) make the argument that the various novel food processing techniques reported by the researchers over this period all reflected improvements upon the methods from which they were adapted.

Jesmer et al. (2018) studied the migratory behavior of translocated populations of bighorn sheep and moose. They hypothesized that if adaptive migration routes that tracked high quality forage (“green‐wave surfing”) were learned, then newcomers should not initially exhibit this behavior in an unfamiliar environment. They found that newly translocated bighorn sheep exhibited lower rates of migration compared with well‐established historical populations, and that their resulting foraging patterns were significantly less well‐matched to the availability of food. Furthermore, in populations of sheep and moose that had populated novel environments 10–110 years previously, green‐wave surfing increased with the length of time since the population was established. Jesmer et al. (2018) thus argue that adaptive knowledge of migration routes is accumulated over long time periods (around 200 years), transmitted between generations via cultural inheritance.

Schofield et al.'s (2017) and Jesmer et al.'s (2018) arguments both make valuable use of historical data spanning multiple generations, which is an essential step toward identifying direct evidence for cumulative cultural evolution in the spontaneous behavior of natural populations. Nonetheless, these claims still depend largely on inferences from circumstantial evidence. Jesmer et al. (2018) draw conclusions about longitudinal within‐population learning effects from cross‐sectional between population data. And Schofield et al. (2017) defend their assumptions about social transmission on the basis of behavioral similarity between closely associated individuals.

However, in spite of the fact that it is possible to criticize the various claims for cumulative culture based on observational data, it should be noted that there are—in all cases—exceptionally good reasons why the researchers in question have proposed these interpretations of their datasets, over and above possible alternatives. The examples of natural behavior which have been ascribed to cumulative cultural evolution (including very early descriptions, as well as more recent analyses) are highly compelling. They have provided a focus for discussion of the possibility of cumulative culture in species other than humans, and have motivated much of the efforts toward systematic experimental evidence, which we consider in the sections to follow.

### 3.2 Studies of adaptive solution‐switching

Some experimental work on capacities for cumulative culture (primarily focused on nonhumans, but also extending to comparisons with human children) has investigated the ease with which individuals will abandon a familiar method in favor of one that is more effective. One of the first studies of this type was carried out by Marshall‐Pescini and Whiten (2008), who presented chimpanzees with a foraging apparatus which could be operated in two alternative ways. “Dipping” was a relatively simple technique which involved inserting a tool directly into the edible contents, but which produced a low yield. “Probing,” by contrast, was a two‐step method which involved using the tool to manipulate the apparatus itself, releasing a catch which then revealed the contents in their entirety. Five chimpanzees that successfully learned the dipping technique were then exposed to demonstrations (by a human experimenter) of the probing technique, but only one individual successfully used this alternative method. Marshall‐Pescini and Whiten (2008) attributed the failure to switch methods to behavioral conservatism, and argued that such conservatism could potentially inhibit cumulative culture in this species.

Subsequent studies following a similar logic have provided mixed results, even considering only other studies with chimpanzees. Some have reported failure to adopt methods that were more profitable than those already in their repertoire (e.g., Bonnie et al., 2012; Hopper, Schapiro, Lambeth, & Brosnan, 2011; Hrubesch, Preuschoft, & van Schaik, 2009; Price, Lambeth, Schapiro, & Whiten, 2009). Others, in contrast, have reported the relinquishing of established techniques in favor of the more advantageous alternative (e.g., Hopper, Kurtycz, Ross, & Bonnie, 2015; Van Leeuwen, Cronin, Schütte, Call, & Haun, 2013; Yamamoto, Humle, & Tanaka, 2013). Davis, Vale, Schapiro, Lambeth, and Whiten (2016) further contributed to this line of research, finding evidence for context‐dependence in chimpanzees' conservatism.

However, regardless of whether interpretations come down in favor of conservatism or flexibility, it should be noted that any inferences regarding the capacities or otherwise for cumulative culture may in any case rest on flawed assumptions. Such inferences are drawn on the basis that cumulative culture depends on individual‐level behavioral modification. This is made explicit in some publications, for example,

cumulative culture *ultimately requires* the ability to change established behaviours in order to adopt more efficient or productive ones; that is, in order to upgrade solutions, *an individual must possess* the behavioural flexibility to relinquish, modify and build on prior solutions (Davis et al., 2016, p1, italics added).

However, this may not be strictly true. Even if individuals cannot switch to a novel solution to a problem already solved in an alternative way, cultural change in the direction of improvements could still occur. For example, it could arise from a systematic bias in deviations from perfect transmission fidelity in the direction of increased effectiveness (e.g., as a result of selective copying). Alternatively, it could also arise from a combination of population‐level variation in behavior (e.g., if there is some inaccuracy associated with social learning), and preferential adoption of more effective variants (e.g., as a result of social learning biases). Consider, for example, the model described by Henrich (2004) which demonstrates how skill level can increase as a result of a combination of success‐biased trait adoption, and variation in skill level generated by imperfect transmission. Therefore, it does not follow that cumulative culture is precluded by any difficulties, at the individual level, with switching to more effective solutions.

Furthermore, it also does not follow that positive results from these studies are indicative of capacities for cumulative culture. Simply showing that an individual can socially learn an effective alternative behavior provides no indication that such a solution could be built up, over more than one generation of transmission, starting from a baseline of completely naïve exploration of the apparatus or problem space.

### 3.3 Closed group studies

Closed group studies, in contrast, have greater potential for investigating whether a task solution can be built up cumulatively over a series of learning steps. These designs involve groups of participants with fixed membership (hence “closed”), and generally allow for multiple attempts at a specified task or problem (thus creating opportunities for social learning between members of the group, who have access to information about others' attempts).

Closed group designs have been used to investigate questions about when and how adult human participants make use of social information, and whether this generates increasingly effective solutions to the task or problem in question (e.g., Derex, Beugin, Godelle, & Raymond, 2013; Derex & Boyd, 2016; Mesoudi, 2008). However, this approach has also been employed as a means to evaluate capacities for cumulative culture in both nonhumans and human children. One key study adopting this approach was carried out by Dean, Kendal, Schapiro, Thierry, and Laland (2012), who aimed to investigate capacities for cumulative culture in preschool children, chimpanzees, and capuchin monkeys. Dean et al. (2012) introduced a puzzle‐box apparatus to groups of these different subject populations. Rewards of varying value could be obtained from the apparatus, with the reward value increasing with the length of the sequence of actions required to access it. The groups of children were generally successful in accessing the rewards, including those involving the longest action sequences. Furthermore, transmission of these solutions was correlated with measures of imitative behavior and teaching, suggesting they were being socially transmitted (although no asocial baseline success rates were reported). In contrast, in the groups of chimpanzees and capuchins, the higher‐level solutions were very rarely found, and there was little evidence of any social learning. The authors concluded that cumulative culture must depend on teaching, imitation, and prosocial tendencies, and that these are present in humans but “absent or impoverished” (p. 1117) in chimpanzees and capuchins.

We return later to the problem of interpreting negative results (which in the context of assessment of capacities for cumulative culture usually means an absence of evidence of increasing success). However, in closed group designs, as with studies of adaptive solution‐switching described above, even positive results suffer from problems of interpretation. The main reason for this is that it is very difficult to separate the influence of direct personal experience with the task or problem from the effects of exposure to social information. As we expect success to increase with increasing experience, as a result of asocial learning processes such as trial‐and‐error, it is not surprising that closed groups are typically found to home in on effective strategies. But it remains unclear whether this would translate into shortcuts to achieving this same level of proficiency for newcomers to the group, as a consequence of exposure to social information.

It should be noted that in the case of Dean et al.'s (2012) study, the interpretation was based largely on the negative results from nonhuman primates, rather than the positive results from children (i.e., Dean et al. concluded that the results showed that nonhuman primates did not have capacities for cumulative culture, rather than that the young children did). However, other studies using this method have sometimes read more into the discovery of complex or effective methods in closed groups.

For example, McGuigan et al. (2017) presented groups of 3‐ to 4‐year‐old children with a puzzle‐box apparatus which, similar to the one used by Dean et al. (2012), contained rewards of a range of values, and could be manipulated in multiple ways, with more complex manipulations associated with the higher value rewards. Discovery of the more advanced and rewarding methods generally followed discovery of the simpler variants, and there was some evidence of social transmission of particular techniques (e.g., within‐group similarity). This led the authors to conclude that the children showed evidence of capacities for cumulative culture. Nonetheless, even if the more advanced techniques were indeed socially learned, it is unclear how much personal familiarity with the apparatus was needed to acquire these. It is therefore not possible to tell whether naïve individuals joining the group would experience shortened learning times, or indeed whether learning from such newcomers alone could facilitate the learning of a subsequent generation of learners further still.

### 3.4 Generational replacement designs

Questions regarding the effects of generational turnover are directly addressed in studies involving the successive replacement of experienced individuals with naïve participants. Generational replacement designs are sometimes characterized as two distinct approaches (linear transmission chains and replacement methods, e.g., Mesoudi & Whiten, 2008). However, it should be noted that the archetypal designs referred to by these labels differ in more than one potentially relevant dimension, and design types which combine elements of each can—and do—exist. Broadly, a typical linear transmission chain design (e.g., Mesoudi, Whiten, & Dunbar, 2006) would involve unidirectional information transfer only (i.e., only down generations, never up), exposure to information from only one potential cultural parent, and no temporal overlap in the roles played by members of different cultural generations (e.g., learner/teacher, or observer/player). In contrast, a typical replacement design (e.g., Caldwell & Millen, 2008) usually allows for bidirectional transfer of information (i.e., the responses of members of one generation can potentially be influenced by the activities of members of subsequent generations), each participant is exposed to information from multiple potential cultural parents, and there is some degree of temporal overlap in the roles played by members of different cultural generations.

Regardless of these differences, the key feature that unites generational replacement designs is the scheduled turnover of participants, and the resulting equivalence of opportunity across generations (e.g., in terms of time constraints). In this way, these methods effectively test whether members of later generations really are benefitted, relative to their predecessors, by virtue of their exposure to higher quality social information, rather than simply increased exposure.

To describe generational replacement designs more precisely, essentially these involve recruiting sets of *n* participants, [*G*_1_, *…*, *G*_*n*_], with participants *G*_*j*_ placed at a particular point (their “generation”), *j*, in a sequence of learners. The chain starts with *G*_1_ participants attempting the experimental task, with their behavior generating some information *I*_1_. The next participant in the chain, *G*_2_, is exposed to this information *I*_1_, with *G*_2_'s behavior then generating *I*_2_, and so on up to participant *G*_*n*_ and information *I*_*n*_. Through analysis of [*I*_1_, *…*, *I*_*n*_], it is possible to study how behaviors change over multiple generations of transmission. If, for example, *I*_*j*_ typically represents higher task success as *j* increases, then this could be evidence of a ratchet effect. Using these types of design, studies with adult humans have found that participants in later generations tend to perform better than those in earlier generations faced with the same task (e.g., Caldwell & Millen, 2008; Wasielewski, 2014; Zwirner & Thornton, 2015), consistent with a process of cumulative cultural evolution.

Generational replacement designs therefore do have the power to identify potential for cumulative culture, given a positive effect of increasing task success over experimental generations. As a result, these methods are now being employed in studies of nonhumans and human children, as a means of investigating whether particular populations display capacities for cumulative culture. For example, Sasaki and Biro (2017) used a generational replacement design to study the efficiency of routes chosen by homing pigeons, finding that later generations used shorter routes, compared with those of their experimental predecessors.

Also using a transmission design, Tennie, Walter, Gampe, Carpenter, and Tomasello (2014) presented children with a task in which they were required to transport rice from one location to another, using any of a set of tools and materials provided by the experimenter. By studying chains of 4‐year‐olds, who were able to observe the previous child's attempt before embarking on their own, Tennie et al. (2014) found that these chains were not able to improve on the efficiency of solutions that the children in the first generation (i.e., those with no exposure to social information) spontaneously adopted as a means to approach the task. Reindl and Tennie (2018) also failed to find evidence of cumulative culture in 4‐ to 5‐year‐old children using a task and design adapted from Caldwell and Millen's (2008) study of tower building in adult human participants. Children were shown the end state of previous children's attempts, but in spite of this, tower height did not increase over generations within the chains.

## 4 LIMITATIONS OF ESTABLISHED METHODS OF ASSESSING CAPACITIES FOR CUMULATIVE CULTURE

Although generational replacement designs may be more suitable for demonstrating evidence of capacities for cumulative culture, compared with adaptive solution‐switching and closed group methods, there are surprisingly few generational replacement studies involving nonhumans or young children. It is likely, however, that the significant logistical challenges associated with such methods are largely responsible for their limited use, especially in relation to testing individuals from special populations (i.e., groups defined more narrowly than the general population, Delaney Shields, 2018). Generational replacement designs usually involve both spatial and temporal coordination between participants, or at the very least coordination of transfer of information between participants. These logistical challenges are almost certainly the reason why the one nonhuman study discussed (Sasaki & Biro, 2017) focusses on pigeons rather than any of the species argued to have shown evidence of cumulative culture in their natural behavior (see previous section). The challenges of running a generational replacement design with chimpanzees, for example, are well illustrated by Horner, Whiten, Flynn, and de Waal's (2006) study of transmission fidelity (not cumulative culture) in chimpanzees. Horner et al. (2006) succeeded in running just two transmission chains of four and five generations each, and yet even these required reparation of multiple links broken due to aggression or noncompliance. Indeed, the high level of control required over the participation and interaction of study subjects may mean that, even where this is possible in principle, for many nonhuman species (and indeed for young children) effective implementation of generational replacement designs is likely to cause unjustifiable stress.

Generational replacement designs also demand large sample sizes, which may limit their usefulness in studying any highly specific participant population, particularly for groups that may only exist in very restricted numbers (e.g., endangered species). These pragmatic considerations relating to sample size are likely the reason why studies of human children have so far been restricted to single age groupings, thus precluding conclusions regarding developmental timeframes, and/or age‐dependent changes in performance.

Taking these factors into account, it is perhaps not surprising that the number of studies of this type remains limited, and that to date there have been no direct comparisons between populations (e.g., children of different ages). Perhaps even more problematic from the point of view of testing capacities for cumulative culture in specific populations, especially those for which there may be some doubt over their ability, is the fact that these complications are also multiplied for designs involving comparisons between experimental conditions. Studies such as those reported in Caldwell and Millen (2008) and Sasaki and Biro (2017) demonstrate proof of principle, but far greater insight can be obtained by establishing the range of conditions under which such effects emerge (e.g., Caldwell & Millen, 2009; Fay, De Kleine, Walker, & Caldwell, 2019; Wasielewski, 2014). However, addressing such questions using multigenerational study designs demands even higher sample sizes, and even greater control over participants' behavior and interactions.

Nonetheless, robust designs involving a range of conditions are likely to be critical in order to establish convincing negative evidence (i.e., lack of capacity for cumulative culture). Failure to identify increases in task success over generations *in a particular context* would not necessarily preclude the possibility of cumulative culture occurring in that population under different circumstances. Furthermore, given sample size requirements, null results could also arise simply from underpowered designs. And yet, for any claim regarding the limits on capacities for cumulative culture, it will be particularly critical to either establish consistent findings across a range of possible contexts, or to identify clear contrasts between different conditions.

## 5 AN ALTERNATIVE METHOD OF ASSESSING CAPACITIES FOR CUMULATIVE CULTURE: INFERRING OUTCOMES OF TRANSMISSION USING DATA FROM INDIVIDUALS

**Figure 1:**
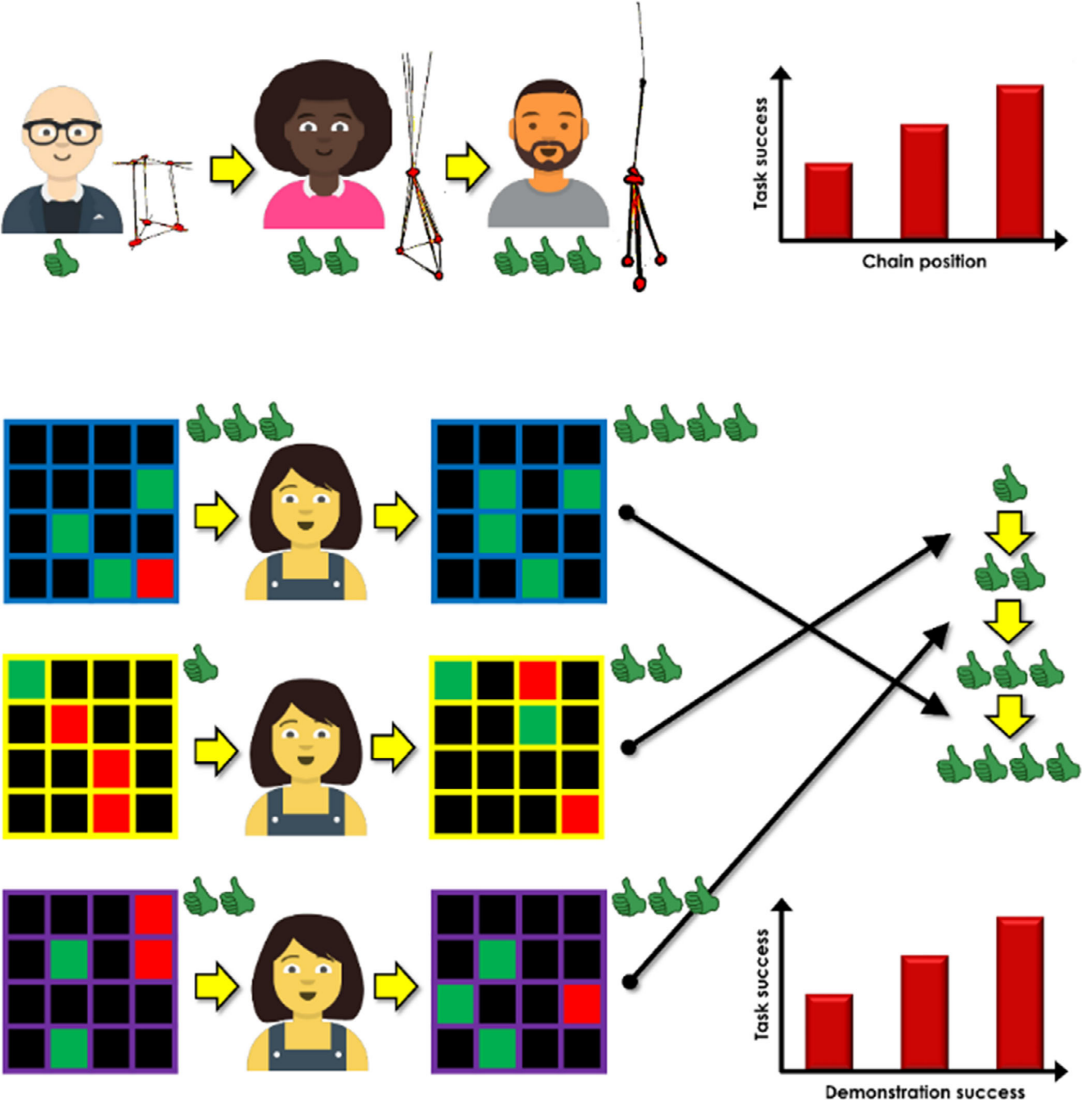
Methods of assessing capacities for cumulative culture. Top: Solution success increases in a chain of multiple individuals attempting a single problem. Below: Solution success increases with demonstration success in single individuals attempting multiple problems. In the examples used, the goal of the participant is to build a tall tower (top), and to find green reward stimuli in a grid search game (below)

We propose that the logistical obstacles discussed above can be largely overcome (or at least considerably reduced) by using an alternative method of assessing capacities for cumulative culture, which combines the strengths of generational replacement designs with the feasibility of simpler methods. For certain types of task, it is possible to infer the outcome of repeated transmission events using data from individuals rather than chains. In order to do this, individuals must be tested on multiple trials, each involving exposure to vicarious information, with that vicarious information reflecting a range of possible task performances. (We use “vicarious information” here to refer to information that is passively—although not necessarily effortlessly—acquired, as opposed to information generated by the individual's own exploration of or interaction with the task.) To use the notation previously established, in this case each single individual *G* would, for example, generate substantially more data than an individual *G*_*j*_ in a linear transmission chain of *n* individuals *G*_1_, …, *G*_*n*_. With regard to cumulative culture, the question of interest is likely to be whether task success increases over successive episodes of social transmission. Therefore, an individual *G* can be exposed to demonstrations of varying success in order to determine whether exposure to high success demonstrations leads to more successful performances. Instead of a single participant *G*_*j*_ in a chain only being exposed to some information *I*_*j−*1_ from the previous participant *G*_*j−*1_, for example, *G* would be exposed to a set of information {*I*_*a*_, *I*_*b*_, *I*_*c*_, …}, with the pieces of information varying in task success and presented as individual experimental trials. In this way it becomes possible to simulate the outcomes of a series of links in a transmission chain, and determine whether task solutions would be subject to a cultural ratchet effect over multiple transmission events (see Figure 1). If, for example, we know how a participant responds to all *N* possible types of information, {*I*_*a*_, *I*_*b*_, *I*_*c*_, …, *I*_*N*_}, then we can simulate their behavior in a chain; if, following some exposure to *I*_*z*_ a participant produces *I*_*x*_, and following *I*_*x*_ produces *I*_*y*_, and so on, we can assess the sequence [*I*_*z*_, *I*_*x*_, *I*_*y*_, …] for a ratchet effect. And, provided we take into account the implementation considerations discussed below, we can do this regardless of the order in which *G* did the trials in which they were exposed to *I*_*x*_, *I*_*y*_, and *I*_*z*_. We provide a concrete example of how this might be put into practice in the section on implementation below.

### 5.1 Methodological precedents

Although such an approach has yet to be applied to the question of cumulative culture specifically, there are comparable methodological precedents within the existing literature, which have been used to answer questions about other cultural evolutionary processes (i.e., not involving ratchet‐like improvement over generations). For example, Griffiths, Christian, and Kalish (2008) used iterated learning methods to investigate human inductive biases. Griffiths et al. (2008) reported two approaches by which this was done: between‐subjects and within‐subjects. The participants' task was to infer hypotheses underlying category membership, based on a small number of either positive (members of the category) or mixed (members and nonmembers) exemplars. The measure of interest was the proportion of trials in which participants selected hypotheses corresponding to particular types of category structure, based on the assumption that there would be a bias toward simpler category structures. In the between‐subjects approach, the category membership rule inferred by a participant was then used to generate the set of stimuli to be presented to the next participant in the chain. In this way it was possible to determine the nature of participants' biases toward certain types of hypotheses. However, as Griffiths et al. (2008) noted, this between‐subjects method depends on the assumption that all learners share the same inductive biases, which may not be the case. In their within‐subjects approach, Griffiths et al. (2008) implemented essentially the same procedure (generating input stimuli based on the hypotheses inferred from the preceding set) but without participant turnover. Therefore, participants were simply exposed to exemplar stimuli drawn according to the category membership rule that they themselves had inferred on the previous trial. Griffiths et al. (2008) highlighted the practical advantage of this approach, which does not require coordinating responses between participants, as each participant generates their own sets of chains. However, they also drew attention to the potential disadvantage that participants' previous responses might influence their subsequent decisions. Griffiths et al. (2008) concluded that there was no evidence of any systematic difference in outcome between the within‐subject and between‐subject designs for their task.

Feher, Ljubicic, Suzuki, Okanoya, and Tchernichovski (2017) used a logically similar design to study the mechanisms underlying the social transmission of bird song in zebra finches. In previous work, Feher, Wang, Saar, Mitra, and Tchernichovski (2009) had reported that wild‐type zebra finch song could develop over several generations of social transmission, starting from the impoverished song of a bird that had been deprived of exposure to species‐typical vocalizations during the sensitive period for song learning. This suggested that the birds were not only able to use the auditory input as a template for the development of their own song, but that they actively transformed this input in the direction of increasingly wild‐type signals. Feher et al. (2017) thus tested whether it was possible for birds that had been raised in isolation to learn wild‐type song through an iterative process of exposure to their own output. They trained juvenile finches using playbacks of their own song, updating these recordings continuously throughout development. Feher et al. (2017) found that birds that received these self‐generated inputs developed wild‐type song as fast as those that had been exposed to the wild‐type song of another individual.

Similar to Griffiths et al. (2008) and Feher et al. (2017), Claidière et al. (2018) wished to compare the outcome of between‐subjects transmission chains with data that had been obtained without transmitting responses between individuals. However, in contrast to the approaches of Griffiths et al. (2008) and Feher et al. (2017), both of whom tracked the performance of individuals over a process of repeated exposure to their own—continually updated—outputs, Claidière et al. (2018) instead used data from a large number of independent trials to simulate the outcome of iterated learning, based on the transition probabilities of different combinations of input and output. In a previous study, Claidière et al. (2014) had trained baboons to reproduce patterns of responses, presented visually on touchscreen computers. By using the baboons' behavioral output as the input for another individual, the researchers created transmission chains which allowed them to document the cultural evolution of systematic structure within the response patterns. Claidière et al. (2018) used data acquired as part of this previous study, which consisted of filler trials using random response patterns (as opposed to the response patterns generated by other individuals as in their previous analysis). They used this data to simulate the outcome of transmission chains, comparing this to the outcome of the real transmission chains as reported in Claidière et al. (2014). The results of the simulations matched the real transmission data well, identifying similar increases over generations in the both the proportions of particular structure types, and the accuracy of reproduction.

### 5.2 Identifying potential for ratcheting from simulated transmission

We propose that an analogous approach could prove hugely beneficial within research on cumulative culture, circumventing the need to coordinate multiple participants. Using individuals as the unit of study, rather than chains, brings a number of important advantages. First, there are logistical benefits, as data collection becomes more straightforward as a result of eliminating the need to transmit responses between subjects. A further practical consideration concerns sample size. Transmission studies generally require a large number of subjects, each of whom produces only a small number of responses. However, using this alternative approach, sample sizes can be reduced in favor of obtaining a larger pool of responses from each subject. Generating a large amount of data at the individual level has the added advantage of allowing for much greater precision regarding individual variation, and between‐group differences. In theory, given enough data, a single individual's response profile can be accurately described, and compared to that of other individuals. At the level of the group, individual data can be aggregated according to any categorization to which those individuals can be classified (e.g., 3 years old vs. 4 years old, or males vs. females), without problems of dependencies between responses generated by members belonging to different categories.

### 5.3 Implementation

As noted previously, in applying this type of method to the question of cumulative culture, the researcher will essentially be looking to establish whether task success would theoretically increase over several generations of transmission. However, as a result of this, there are additional considerations that must be taken into account in order to infer the outcomes of transmission from individual data. Although Griffiths et al. (2008) noted the potential problem of carry‐over effects in their within‐subjects datasets, this is less of a concern for their study (and indeed those of Feher et al., 2017; and Claidière et al., 2018). In these previous studies, the target response on any given trial is dictated by the output used to generate the input stimuli. Each trial therefore essentially represents a fresh problem, with its own particular target response (notwithstanding a degree of dependency between these). However, consider an equivalent design applied to the kinds of tasks that have been used to study cumulative culture in the laboratory (which include: paper aeroplane building and spaghetti tower building (Caldwell & Millen, 2008); creation of load‐bearing devices, (Wasielewski, 2014); generation of a target image using a graphics software package, (Muthukrishna, Shulman, Vasilescu, & Henrich, 2014); and basket creation (Zwirner & Thornton, 2015). Each of these experiments focused on one single problem, which remained constant for all participants, as did the criteria for task success.

Using tasks like these it is not possible to use a participant's responses to a range of potential demonstrations to predict the outcome of transmission, as any assumption of prior naivety to the problem is severely violated. The participant's response will be determined not only by the information to which they are exposed on that particular trial, but also by their prior experience of the task and their exposure to vicarious information on previous trials. Thus it is not possible to determine how a naïve learner would fare when exposed to *only* the information available at a particular point in the chain. Fulfilling the assumption of naivety to the problem is clearly essential if aiming to draw inferences about cumulative culture, as explained previously in relation to the strengths of generational replacement designs compared with other methods. It is a signature feature of cumulative culture that members of later generations are advantaged relative to earlier ones as a result of the quality of the information they encounter, despite each generation having approximately matched learning opportunities (e.g., comparable exposure times).

Nonetheless, we believe that it is possible to draw inferences about the potential for cumulative culture, using methods similar to those of Griffiths et al. (2008) and Claidière et al. (2018), although this depends on using experimental tasks that are rather different to those used in the literature to date. To avoid carry‐over effects, multiple problems of the same type (each with their own independent solution) must be used, rather than relying on a single problem with a fixed solution space. Of course, it is important that these different problems can be regarded as fully equivalent to one another, despite their different solutions. In particular, the measure of success, or task score, must be directly comparable between problems. This is likely to necessitate a relatively abstract task, which may involve sacrificing a degree of ecological validity. Although we return to the issue of the limitations of the approach we propose (see section below) there are also advantages to using an abstract task, as this allows great potential for manipulation of variables potentially affecting task difficulty and constraints on transmission (e.g., size of problem space, demonstration exposure time, or interference effects). Furthermore, it would be theoretically possible to produce a task that was completely open‐ended, in the sense of having an unbounded problem space which could extend in response to a participant's performance.

As an illustrative example, here we describe one potential method by which this approach to studying cumulative culture could be implemented. Figure 1 (lower section) depicts a simple stimulus selection task. The participant's task is to search for rewarded stimuli (of which there are four in the examples shown) hidden within the arrays (which each consist of 16 stimuli in total). On average, a naïve individual exploring the grid without any information about the reward distribution is likely to find only one of the rewarded stimuli. However, another individual who has the benefit of exposure to this chance‐level performance can in theory improve upon this score if faced with the same problem. Optimal use of the information will result in retention of the rewarded stimulus whose location has been revealed, and potential discovery of additional rewarded stimuli as a consequence of avoidance of the locations of the unrewarded stimuli. Over multiple transmission events between agents making optimal (or close to optimal) use of the information to which they are exposed, scores will tend to increase. Members of later generations would therefore benefit, relative to their predecessors, finding more of the rewarded stimuli.

In order to infer the likely outcome of transmission, without actually passing information between participants, individuals simply need to complete multiple trials representing a range of possible scores. The trials must use problems of the same general structure (e.g., find four rewarded stimuli within a 16‐item array) but carry‐over effects can be avoided by basing each trial on a novel problem with its own independently generated reward distribution. While we envisage that such “demonstration” trials would typically be randomly generated, these could be validated against, or even derived from, real participant responses, to provide some reassurance regarding the ecological validity of the stimuli. The information can be provided to participants in a truly “social” context (e.g., by using a trained conspecific demonstrator, or a human experimenter) but in some instances it might be sufficient to provide the information in the form of task cues in the absence of a demonstrator. It is perhaps worth noting that no physical “demonstrator” was present during subjects' exposure to the input stimuli in any of the methodological precedent studies cited above (Claidière et al., 2018; Feher et al., 2017; Griffiths et al., 2008). Nonetheless, presenting information in a social context may be desirable under some circumstances. For example, when testing young children it may facilitate their understanding of the task if an adult experimenter performs the demonstration. Indeed, given the flexibility of the method we propose, and its abstract structure, the issue of information source is a perfectly tractable empirical question, and therefore it would be possible to determine in advance which mode of presentation would be preferable (e.g., see Renner, Atkinson & Caldwell, under review).

Furthermore, it should be noted that this approach would not preclude the possibility of studying between‐subjects transmission in the same way, for example, as a means of validating results obtained using the within‐subjects designs (similar to the three methodological precedents discussed previously). A between‐subjects design will inevitably entail a number of logistic challenges which would not apply to an equivalent within‐subjects approach. However, any design that permits remote coordination of responses between participants (rather than real‐time in‐person interaction) in itself significantly reduces many of the difficulties associated with established methods of studying cumulative culture. Reinforcing this point, in a recent study Saldana, Fagot, Kirby, Smith, and Claidière (2019) ran cultural transmission chains with both nonhuman subjects (baboons) and human children, using a computer‐based task that involved remote transmission of responses between participants.

### 5.4 Analysis and interpretation

It should be emphasized that the preferred approach to the analysis and interpretation of datasets generated in this way is likely to depend on the researcher's focus, and the constraints under which cultural evolution is assumed to operate. We can envisage situations in which task performance might not permit ratcheting under assumptions of linear transmission (i.e., unidirectional from a single cultural parent), but could potentially support cumulative culture in certain population structures (e.g., see Henrich, 2004; Powell, Shennan, & Thomas, 2009). If assuming linear transmission, participants must be shown to outperform demonstrations of a range of success levels. However, if assuming (with some justification) that cultural evolution is likely to involve exposure to multiple demonstrations within a single generation, this allows greater tolerance of suboptimal performances. Given a degree of success‐bias and/or selective copying, ratcheting over generations could still occur even if participants did not outperform demonstrations, based on a single exposure, at multiple levels of success. Indeed it would be possible to evaluate this empirically using designs involving exposure to more than one demonstration trial per problem.

In all cases, however, we suggest the following minimal criteria for judging the potential for ratcheting: (a) the participant(s) must—at the very least—outperform a chance‐level demonstration (i.e., equivalent to the performance of a naïve individual not exposed to any problem‐relevant information); and (b) performance following an above‐chance level demonstration should be significantly better than performance following a chance‐level demonstration. The first criterion ensures that participants' performance does indeed exhibit evidence of some benefit of accumulated experience, as opposed to simply a regression to the mean following an unusually ineffective demonstration. The second criterion ensures that participants gain increased benefit from social information of increasing quality. It may be possible to derive the naïve baseline statistically, but this could also be determined empirically from real responses of participants attempting problems without exposure to relevant information. An additional advantage of this method is that it generates datasets (consisting of a collection of input–output relationships), that can be readily fed into simulations which can provide qualitative insights into the outcomes of repeated transmission events. A simulation‐based approach could also extend to comparing outcomes generated under different transmission conditions (e.g., see Claidière et al., 2018), allowing direct investigation of questions about the effects of different population structures.

### 5.5 Limitations

While the method that we have proposed may bring considerable advantages in terms of flexibility and practicality, we fully acknowledge that it is not without drawbacks. As previously noted, this approach demands a relatively abstract task (such that multiple different problems of the same type can be generated, all appropriately equivalent to one another, but also totally independent in their specific solutions), and this is likely to compromise ecological validity. The specifics of the research question and population under investigation will dictate whether or not this is a critical issue. For certain populations, it may be unrealistic to expect subjects to demonstrate their full learning potential in a context that is perceptually and behaviorally quite far removed from the context in which such abilities might be used in their natural environment. However, up to a point, it may be possible to tailor superficial features of the presentation of the task while preserving the logical structure.

The task structure that we suggest might also prove problematic from the perspective of some definitions of cumulative culture, which require that the transmitted behavior must be something that no single individual could invent alone (e.g., see Miton & Charbonneau, 2018). However, as noted previously, the abstract nature of the task means that problems could be (at least in principle) open‐ended in their difficulty, whereas this has typically not been true of tasks previously used in experimental studies of cumulative culture. However, it is certainly true that the task design that we propose does not effectively capture insightful innovation, which could be a key driver of human cumulative culture. We consider this an important future challenge for this area of research.

It should also be noted that although the approach we propose offers some potential to investigate variables of group size and structure, it is still only possible to consider unidirectional information transmission. Thus any investigation into the effects of population variables would be limited to manipulations of the type and quantity of information available to the participant. Any process involving bidirectional communication, including feedback from information receivers, interactions between participants, or coordination of responses, cannot be addressed using this method.

A further issue, which again may or may not represent a serious flaw depending on the research question, is the need for subjects to learn the predictive relevance of the information they receive. Clearly, in order to have any chance of making effective use of the information, an individual must either understand the link, or form an association, between the vicariously experienced task cues indicating positive and negative outcomes, and the reward value of the corresponding stimuli from which they can make their own selections. Furthermore, these within‐problem predictive relationships must be grasped, in spite of the arbitrary nature of between‐problem relationships. While this understanding can be effectively scaffolded in children, by using understandable contextual framing, and even explicit explanations for those old enough to understand language, scaffolding of this kind is unlikely to be possible for nonhuman populations. In this case, the predictive value of the information will almost certainly need to be learned from experience, which in most cases would be achieved through training of the relevant associations.

The requirement to train subjects to recognize the relationship between the information and test elements of a particular problem may not be a problem for certain research questions. If the researcher wishes to investigate, for example, the extent to which the information can be used strategically to outperform the input information received, then it may not matter how the relevant associations were formed originally. Dependence on training is also unlikely to be a problem if the question of interest involves comparing task performance under different conditions to determine whether, and how, these differentially constrain performance. However, for researchers interested in understanding how social information is spontaneously used (e.g., as a consequence of prior “real world” experience, or instinctive biases) this is probably not an appropriate method.

## 6 CONCLUSION

Despite great interest in questions regarding capacities for cumulative culture, empirical investigation has been impeded by the methodological difficulty of studying a population‐level phenomenon using experimental methods. The complex designs of established lines of research have limited the scope for expansion to studying novel populations, or for asking more detailed questions regarding the constraints on cumulative cultural outcomes. In the current review, in addition to providing an overview of some of these existing methods, we have also proposed an alternative approach which may have potential to open up promising avenues of investigation. By predicting the outcomes of transmission from data collected at the individual level, experimental designs would become much more feasible. The ability to make inferences about the potential for cumulative culture without the need for spatial and temporal coordination between participants is likely to prove particularly valuable for studying populations that may be sensitive, or simply difficult to access. Furthermore, removing dependencies between responses provided by different individuals ought to bring benefits for the precision and reliability of conclusions, as well as reducing sample size requirements.

Thus, we envisage that methods such as the one proposed here will allow researchers to answer previously intractable questions about capacities for cumulative culture. Such questions might concern, for example, the conditions under which we might expect to see cumulative learning benefits accruing over transmission in a wide range of different species (e.g., by manipulating the persistence of social information cues to remove cognitive storage constraints). Studying the development of children's capacities for cumulative culture would also become much more straightforward, which would permit insights into any concomitant cognitive advances which appeared to predict shifts in the efficiency with which children could use information acquired from observation. There is also great future potential for research involving adult humans as well, as reduced sample size requirements, and logistical ease, will make designs involving multiple conditions more attainable.

In conclusion, we hold an optimistic view regarding the progress that can be made in studying capacities for cumulative culture. A great deal has been achieved already using established methods, and novel approaches offer great promise for further expanding this field.

## CONFLICT OF INTEREST

The authors have declared no conflicts of interest for this article.

## AUTHOR CONTRIBUTIONS

Christine Caldwell: Conceptualization, lead; writing original draft, lead; writing review and editing, lead. Mark Atkinson: Conceptualization, supporting; writing original draft, supporting; writing review and editing, supporting. Kirsten Blakey: Conceptualization, supporting. Juliet Dunstone: Conceptualization, supporting. Donna Kean: Conceptualization, supporting. Gemma Mackintosh: Conceptualization, supporting. Elizabeth Renner: Conceptualization, supporting. Charlotte Wilks: Conceptualization, supporting.

## RELATED WIREs ARTICLES

https://doi.org/10.1002/wcs.1196

https://doi.org/10.1002/wcs.1288

https://doi.org/10.1002/wcs.14
